# Alsin Related Disorders: Literature Review and Case Study with Novel Mutations

**DOI:** 10.1155/2014/691515

**Published:** 2014-09-14

**Authors:** Filipa Flor-de-Lima, Mafalda Sampaio, Nahid Nahavandi, Susana Fernandes, Miguel Leão

**Affiliations:** ^1^Department of Pediatrics, Hospital Pediátrico Integrado, Centro Hospitalar de São João, Alameda Prof. Hernâni Monteiro, 4200-319 Porto, Portugal; ^2^Faculty of Medicine, University of Porto, Alameda Prof. Hernâni Monteiro, 4200-319 Porto, Portugal; ^3^Unit of Pediatric Neurology, Hospital Pediátrico Integrado, Centro Hospitalar de São João, Alameda Prof. Hernâni Monteiro, 4200-319 Porto, Portugal; ^4^Centogene AG, Schillingallee 68, 18057 Rostock, Germany; ^5^Department of Genetics, Faculty of Medicine, University of Porto, Alameda Prof. Hernâni Monteiro, 4200-319 Porto, Portugal

## Abstract

Mutations in the *ALS2* gene cause three distinct disorders: infantile ascending hereditary spastic paraplegia, juvenile primary lateral sclerosis, and autosomal recessive juvenile amyotrophic lateral sclerosis. We present a review of the literature and the case of a 16-year-old boy who is, to the best of our knowledge, the first Portuguese case with infantile ascending hereditary spastic paraplegia. Clinical investigations included sequencing analysis of the ALS2 gene, which revealed a heterozygous mutation in exon 5 (c.1425_1428del p.G477Afs*19) and a heterozygous and previously unreported variant in exon 3 (c.145G>A p.G49R). We also examined 42 reported cases on the clinical characteristics and neurophysiological and imaging studies of patients with known ALS2 gene mutations sourced from PubMed. This showed that an overlap of phenotypic manifestations can exist in patients with infantile ascending hereditary spastic paraplegia, juvenile primary lateral sclerosis, and juvenile amyotrophic lateral sclerosis.

## 1. Introduction

Three apparently distinct disorders involving retrograde degeneration of the upper motor neurons of the pyramidal tracts seem to be caused by mutations in the ALS2 gene, which provides instructions for making a protein called Alsin. They comprise a clinical continuum from infantile ascending hereditary spastic paraplegia (IAHSP) (OMIM number 607225), to juvenile forms without lower motor neuron involvement, namely, juvenile primary lateral sclerosis (JJPLS) (OMIM number 606353), and to forms with lower motor neuron involvement, namely, autosomal recessive juvenile amyotrophic lateral sclerosis (JALS) (OMIM number 205100) [1, 2]. There is no available data on the prevalence of ALS2 related disorders. However, they are probably currently underdiagnosed, even if they have been described in individuals from a variety of ethnic backgrounds, mainly from the Mediterranean [1].

All the patients are homozygous or heterozygous compounds for ALS2 mutations [1]. To date, a total of 45 patients with known mutations in the ALS2 gene have been described, but the phenotype-genotype correlation remains unclear [2]. In the present study, we describe the clinical and genetic features of a 16-year-old boy with IAHSP from Northern Portugal (Table 1).

## 2. Case Report

The patient was born after a twin pregnancy from nonconsanguineous parents and the pregnancy included maternal hemorrhage in the second trimester. Delivery was at the 36th week of gestation by Cesarean section. The twins were dizygotic twins and the patient's twin sibling is healthy. His 42-year-old mother is healthy and his father died at the age of 35 after a car accident, without any signs of a neurological disorder. The boy acquired cephalic control at three months and started to sit unaided at six months, crawl at nine months, and walk with support at 10 to 11 months. Stiffness of the lower limbs and tiptoeing with hyperactive deep tendon reflexes were noticed at the age of three and scissoring gait started during his fourth year. He was never able to walk without support and underwent Achilles tenotomy at the ages of three and five. An ascending progression of motor difficulties was observed, with spasticity becoming evident in the upper extremities after the age of six. Muscle atrophy in the lower limbs was evident after the age of seven and he was wheelchair bound at the age of eight. Sphincter incontinence started at the same time and he developed supranuclear bulbar palsy, with progressive dysarthria. MRI, electromyography, and nerve conduction studies at that age were normal. Anarthria was evident at the age of 13. At the age of 14, there was clinical worsening and since then he has had bilateral limitation of horizontal eye movements, dysphagia when drinking liquids, chewing difficulties, severe drooling, and paroxysms of laughter. Cognitive function is still normal at the age of 16.

## 3. Material and Methods

DNA was extracted from a peripheral blood sample from the patient, his mother, and twin brother. All 34 exons of the ALS2 gene were analysed by PCR and sequencing of both DNA strands of the entire coding region was carried out, including the highly conserved exon-intron splice junctions.

We also reviewed all cases of ALS2 related disorders with known ALS2 gene mutations and detailed clinical, neurophysiological, and imaging data that have so far been reported in PubMed. Continuous variables with asymmetric distribution are described by medians (minimum to maximum) and categorical variables are described by absolute and relative frequencies. To compare the three phenotypes (IAHSP, JALS, and JPLS) we used the Kruskal-Wallis test if the variables were continuous and the Monte Carlo test if they were categorical. The statistical analysis was performed using SPSS v.20 (IBM, USA) and *P* values of less than 0.05 were considered significantly different.

## 4. Results and Discussion

Our patient displays a clinical picture that is highly suggestive of ALS2 related disorder. This case study presents evidence of previously unreported heterozygous variants in exon 5 (c.1425_1428del p.G477Afs*19) and exon 3 (c.145G>A p.G49R).

To date, case studies of 45 patients with ALS mutations have been reported. Four patients with JALS were excluded because a detailed clinical description was not available [3]. The clinical characteristics and neurophysiological and imaging studies of the remaining 41 cases, plus our case study, are summarized in Table 2. Of these, 21 (50%) of the patients were classified as having an IAHSP phenotype, 17 (40.5%) had a JALS phenotype, and four (9.5%) had a JPLS phenotype. Median age at onset of walking loss, upper limb involvement, speech impairment, and becoming wheelchair bound was similar between the three groups.

The heterozygous variant in exon 5 (c.1425_1428del p.G477Afs*19) creates a shift in the reading frame, starting at codon 477. The new reading frame ends in a stop codon 18 positions downstream, which is very likely to result in truncated protein or loss of protein production. Therefore, it is very likely to be a disease causing mutation. A small deletion in this region (c.1427_1428delAG), which also causes a frameshift, has previously been described as disease causing for ALS2 [4]. The other unreported heterozygous variant was found in exon 3 (c.145G>A p.G49R), which is located in a moderately conserved amino acid, with moderate physiochemical differences between the amino acids glycine and arginine. Polyphen-2, SIFT, and MutationTaster predict that this variant is probably damaging. This variant in exon 3 was also found in our patient's twin brother and their mother, who were both healthy. It was impossible to test his father because he was dead.

Despite the limited number of patients reported in the literature with known ALS2 mutations and considering the bias related to the age, the majority of clinical characteristics were similar between both groups. Because all the families reported to date have had different ALS2 mutations, it is impossible to draw any genotype-phenotype correlation.

## 5. Conclusions

Despite the limited information about clinical characteristics, patients with IAHSP, JALS, and JPLS may present with different phenotypes that overlap.

## Figures and Tables

**Table 1:** Mutations in ALS2 related disorders.

Patient	Exon/intron	Mutation	Predicted protein	Phenotypic classification	References
1	Intron 24	c.3836+1G>T	p.k1234fs∗3	IAHSP	Racis et al., 2014 [5]

2	Intron 9	c.2000-2A>T	p.E724fs∗32	IAHSP	Herzfeld et al., 2009 [6]

3	Exon 9	c.1825_1826ins5	p.E609fs∗9	IAHSP	Sztriha et al., 2008 [7]
4	Exon 13	c.2529G>T	p.G1177∗	IAHSP	

5, 6	Exon 10	c.2143C>T	p.Q715∗	IAHSP	Verschuuren-Bemelmans et al., 2008 [8]

7, 8	Exon 4	c.467G>A	p.C156Y	IAHSP	Eymard-Pierre et al., 2006 [9]

9, 10	Exon 18	c.2992C>T	p.R998∗	IAHSP	Devon et al., 2003 [10]

11	Exon 32	c.4844delT	p.I331fs335	IAHSP	Gros-Louis et al., 2003 [11]

12–17	Exon 4	c.1130delAT	p.I331fs335	IAHSP	Eymard-Pierre et al., 2002 [12]
	Exon 13	c.2660delAT	p.N845fs858	IAHSP	
	Exon 6	c.1471_1480del10	p.V491Gfs∗3	IAHSP	
	Exon 22	c.3742delA	p.M1206∗	IAHSP	

18–20	Exon 5	C.1548delAG	p.T475Tfs∗70	IAHSP	Hadano et al., 2001 [4]

21	Exon 5 Exon 3	c.1427_1428del c.145G>A	p.G477Afs∗19 p.G49R	IAHSP	Our study

22-23	Exon 4 Exon 14	c.299G>T c.2580-2A>G	p.S100I	JALS JALS	Luigetti et al., 2013 [13]

24-25	Exon 22	c.3565delG	p.V1189WfsX19	JALS	Shirakawa et al., 2009 [2]

26	Exon 4	c.553delA	p.T185LfsX5	JALS	Kress et al., 2005 [14]

27–38	Exon 3	c.138delA	p.A46AfsX5	JALS	Hadano et al., 2001 [4]

39–41	Intron 17	c.2980-A>G	p.T993fs∗7	JPLS	Mintchev et al., 2009 [15]

42	Exon 6	c.1619G	p.G540E	JPLS	Panzeri et al., 2006 [16]

**Table 2:** Summary of the characteristics of 42 patients with known ALS2 gene mutations.

Patient	Age	Origin	Motor development by 1 year	Age at onset	Loss of walking	Upper limb involvement	Bulbar involvement	Speech impairment	Ocular movements	Wheelchair bound	EMG	Evoked potentials	Brain imaging	Phenotypic classification	References
1	17 y	Italy	Ab	12 mo	NA	8 y	8 y	Disarthria at 8 y, Anarthria at 11 y		8 y	Ab	SSEP ab	Ab	IAHSP	Racis et al., 2014 [5]

2	7 y	Germany	Ab	18 mo	<7 y	<7 y	7 y		N	7 y			Ab	IAHSP	Herzfeld et al., 2009 [6]

3	11 y	Hungary	Ab	10 mo	NA	2 y	5 y	No	N	11 y	N	Motor ab	N	IAHSP	Sztriha et al., 2008 [7]
4	6 y	Hungary	Ab	<1 y	NA	No	5 y	No	N	5 y			N	IAHSP	

5	13 y	The Netherlands	Ab	8 mo	NA	3 y	5 y	Anarthria at 13 y	N	13 y	N	MEP Unobtainable	N	IAHSP	Verschuuren-Bemelmans et al., 2008 [8]
6	8 y	The Netherlands	Grossly N	18 mo	NA	Yes	4 y	No	N	No	N	MEP Unobtainable	N	IAHSP	

7	22 y	Turkey	Ab	1 y	12 y	12 y	16 y	No		12 y				IAHSP	Eymard-Pierre et al., 2006 [9]
8	20 y	Turkey	Ab	1 y	10 y		12 y	No		10 y	N	Motor ab	Ab	IAHSP	

9	9 y	Bukhari Jewish	N	1-2 y	NA	2 y	3 y	Dysarthria at 9 y		No				IAHSP	Devon et al., 2003 [10]
10	6 y	Bukhari Jewish	N	14 mo	6 y	6 y	6 y	Dysarthria at 6 y		No	N		N	IAHSP	

11	12 y	Pakistan	Ab	18 mo	12 y		<12 y	Anarthria at 12 y		12 y				IAHSP	Gros-Louis et al., 2003 [11]

12	36 y	Algeria		1 y	NA	<7 y	13 y	Dysarthria at 13 y	N		N	MEP and SSEP abnormal	Ab	IAHSP	Eymard-Pierre et al., 2002 [12]
13	31 y	Algeria		1 y	NA	<7 y	13 y	Dysarthria at 13 y	N		N	MEP and SSEP abnormal		IAHSP	
14	24 y	Algeria		1 y	NA	<7 y	13 y	Dysarthria at 13 y	N		N	MEP and SSEP abnormal		IAHSP	
15	18 y	France		1.5 y	4 y	6 y	8 y	Dysarthria at 4 y, anarthria at 12 y	Ab		N	MEP and SSEP abnormal	Ab	IAHSP	
16	23 y	Italy		1.4 y	5 y	10 y	12 y	Dysarthria at 10 y, anarthria at 16 y	Ab		N	MEP and SSEP abnormal	Ab	IAHSP	
17	20 y	Italy		1.5 y	4 y	9 y	13 y	Dysarthria at 11 y, anarthria at 18 y	Ab		N	MEP and SSEP abnormal	Ab	IAHSP	

18	14 y	Kuwait	N	14 mo	2 y	9 y	4 y	Dysarthria at 4 y, anarthria at 14 y			N	N	Ab	IAHSP	Hadano et al., 2001 [4]
19	6 y	Kuwait	Ab	11 mo	NA		5 y	Dysarthria at 5 y,	N	No			Ab	IAHSP	
20	2 y	Kuwait	Ab	9 mo	NA									IAHSP	

21	16 y	Portugal	N	3 y	NA	6 y	8 y	Dysarthria at 8 y, anarthria at 13 y	Ab	8 y	N		N	IAHSP	Our study

22	27 y	Italy	N	3 y				Dysarthria at 7 y, anarthria at 14 y			Ab	SSEP N	N	JALS	Luigetti et al., 2013 [13]
23	21 y	Italy	N	6 y							Ab	SSEP N	N	JALS	

24	32 y	Japan	N	13 mo	No		11 y	Dysarthria at 11 y, anarthria at 14 y		No	Ab		N	JALS	Shirakawa et al., 2009 [2]
25	23 y	Japan	N	3 y	No			Dysarthria		No				JALS	

26	32 y	Turkey	Ab	22 mo	16 y	12 y	15 y	18 y		16 y	Ab	Motor ab, SSEP N		JALS	Kress et al., 2005 [14]

27	60 y	Tunisia	N	10 y			10 y				N	Motor N		JALS	Hadano et al., 2001 [4]
28	36 y	Tunisia	N	6.5 y			6.5 y				N			JALS	
29	27 y	Tunisia	N	3.5 y			Yes				N	Motor N, SSEP ab		JALS	
30	22 y	Tunisia	N	6.5 y			6.5 y				N	Motor N		JALS	
31	21 y	Tunisia	N	9 y			9 y				N			JALS	
32	14 y	Tunisia	N	6.5 y			6.5 y				N			JALS	
33	23 y	Tunisia	N	6.5 y			6.5 y				N	Motor N		JALS	
34	28 y	Tunisia	N	3.5 y			Yes				N			JALS	
35	32 y	Tunisia	N	7.5 y			Yes				N	Motor N		JALS	
36	22 y	Tunisia	N	6.5 y			Yes				N			JALS	
37	21 y	Tunisia	N	10 y			Yes				N	Motor N, SSEP ab		JALS	
38	7 y	Tunisia	N	6 y			Yes				N			JALS	

39	55 y	Cyprus	N	2 y	50 y	Yes	3 y		Ab	50 y				JPLS	Mintchev et al., 2009 [15]
40	42 y	Cyprus	N	2 y	2 y	Yes	2 y		Ab	2 y		SSEP N	N	JPLS	
41	16 y	Cyprus	N	2 y	No	Yes	2 y		Ab	No	Ab			JPLS	

42	34 y	Italy	N	2 y	19 y	2 y	6 y	Dysarthria at 6 y, anarthria at 20 y	Ab	34 y	Ab	Motor ab	N	JPLS	Panzeri et al., 2006 [16]

EMG: electromyography; N: normal; Ab: abnormal; NA: not achieved; y: years; mo: months; MEP: motor evoked potentials; SSEP: somatosensory evoked potentials.
